# Characterization of *Aspergillus fumigatus* Extracellular Vesicles and Their Effects on Macrophages and Neutrophils Functions

**DOI:** 10.3389/fmicb.2019.02008

**Published:** 2019-09-04

**Authors:** Jéssica Amanda Marques Souza, Ludmila de Matos Baltazar, Virgínia Mendes Carregal, Ludmila Gouveia-Eufrasio, André Gustavo de Oliveira, Wendell Girard Dias, Marina Campos Rocha, Kildare Rocha de Miranda, Iran Malavazi, Daniel de Assis Santos, Frédéric Jean Georges Frézard, Daniele da Glória de Souza, Mauro Martins Teixeira, Frederico Marianetti Soriani

**Affiliations:** ^1^Centro de Pesquisa e Desenvolvimento de Fármacos, Departamento de Genética, Ecologia e Evolução, Instituto de Ciências Biológicas, Universidade Federal de Minas Gerais, Belo Horizonte, Brazil; ^2^Laboratório de Interação Microrganismo-Hospedeiro, Departamento de Microbiologia, Instituto de Ciências Biológicas, Universidade Federal de Minas Gerais, Belo Horizonte, Brazil; ^3^Laboratório de Biofísica e Sistemas Nanoestruturados, Departamento de Fisiologia e Biofísica, Universidade Federal de Minas Gerais, Belo Horizonte, Brazil; ^4^Laboratório de Micologia, Departamento de Microbiologia, Universidade Federal de Minas Gerais, Belo Horizonte, Brazil; ^5^Lab Circuitos Fisiológicos, Departamento de Fisiologia e Biofísica, Universidade Federal de Minas Gerais, Belo Horizonte, Brazil; ^6^Plataforma de Microscopia Eletrônica Rudolf Barth, Fundação Oswaldo Cruz, Rio de Janeiro, Brazil; ^7^Centro de Ciências Biológicas e da Saúde, Departamento de Genética e Evolução, Universidade Federal de São Carlos, São Carlos, Brazil; ^8^Laboratório de Ultraestrutura Celular Hertha Meyer, Programa de Biologia Celular e Parasitologia, Instituto de Biofísica Carlos Chagas Filho, Universidade Federal do Rio de Janeiro, Rio de Janeiro, Brazil; ^9^Centro de Pesquisa e Desenvolvimento de Fármacos, Departamento de Bioquímica e Imunologia, Instituto de Ciências Biológicas, Universidade Federal de Minas Gerais, Belo Horizonte, Brazil

**Keywords:** extracellular vesicles, filamentous fungus, *Aspergillus fumigatus*, host-pathogen interactions, macrophages, neutrophils

## Abstract

Extracellular vesicles (EVs) has been considered an alternative process for intercellular communication. EVs release by filamentous fungi and the role of vesicular secretion during fungus-host cells interaction remain unknown. Here, we identified the secretion of EVs from the pathogenic filamentous fungus, *Aspergillus fumigatus*. Analysis of the structure of EVs demonstrated that *A. fumigatus* produces round shaped bilayer structures ranging from 100 to 200 nm size, containing ergosterol and a myriad of proteins involved in REDOX, cell wall remodeling and metabolic functions of the fungus. We demonstrated that macrophages can phagocytose *A. fumigatus* EVs. Phagocytic cells, stimulated with EVs, increased fungal clearance after *A. fumigatus* conidia challenge. EVs were also able to induce the production of TNF-α and CCL2 by macrophages and a synergistic effect was observed in the production of these mediators when the cells were challenged with the conidia. In bone marrow-derived neutrophils (BMDN) treated with EVs, there was enhancement of the production of TNF-α and IL-1β in response to conidia. Together, our results demonstrate, for the first time, that *A. fumigatus* produces EVs containing a diverse set of proteins involved in fungal physiology and virulence. Moreover, EVs are biologically active and stimulate production of inflammatory mediators and fungal clearance.

## Introduction

Intercellular communication is a crucial process that occurs from simple organisms, as bacteria, up to complex organisms, including mammals. Communication can happen by direct interaction between cells or by secretion of molecules that act directly to coordinate cellular functions (Nilsen-Hamilton and Hamilton, 1982; Yoon et al., 2014; Yáñez-Mó et al., 2015). Recently, the release of extracellular vesicles (EVs) has been associated with the process of intercellular communication (Yoon et al., 2014). EVs are structures secreted by cells and formed by a lipid bilayer membrane, forming a lumen containing a specific burden of biomolecules, for example, proteins, lipids, polysaccharides and nucleic acids (Yoon et al., 2014). Release of EVs offers simultaneous delivery of many different messenger molecules and may be able to reach sites distant from vesicular origin (Yáñez-Mó et al., 2015).

There is now substantial evidence to suggest that EVs may be key-mediators of the pathogenesis of infections caused by bacteria, parasites, virus and fungi (Joffe et al., 2016). These vesicles may cause host cell death, elicit immune response, and in some cases, confer protection against diseases (Brown et al., 2015). For example, in prokaryotes, molecules able to confer cytotoxicity to host cells, factors responsible for biofilm production and antibiotic resistance have been identified in EVs (Kuehn and Kesty, 2005). In *Escherichia coli*, α-hemolisin fractions were identified inside EVs and were shown to be relevant in red blood cells lysis (Balsalobre et al., 2006). Similarly, *Staphylococcus aureus* EVs may induce cytotoxicity and are able to induce apoptosis *in vitro* cells (Gurung et al., 2011). *Mycobaterium tuberculosis* EVs are able to stimulate the cytokine and chemokine *in vitro* production, and favor the pathogen infection *in vivo* model (Prados-Rosales et al., 2011).

It has been demonstrated that fungi are able to produce biologically active EVs under culture and during infection (Rodrigues et al., 2007; Albuquerque et al., 2008; Gehrmann et al., 2011; Vallejo et al., 2011; Silva et al., 2014; Bitencourt et al., 2018; Ikeda et al., 2018). The characterizations of fungal EVs revealed great variety of molecules with biological function as lipids, polysaccharides and nucleic acids. Besides that, it was also identified proteins known to participate in virulence, cellular metabolism, signal transduction, and nuclear and structure proteins (Albuquerque et al., 2008; Rodrigues et al., 2008; Oliveira et al., 2010; Vallejo et al., 2011; Silva et al., 2014, 2015; Brown et al., 2015; Gil-Bona et al., 2015; Vargas et al., 2015; Ikeda et al., 2018). For example, *C. neoformans* EVs are able to stimulate immunomodulatory mediators by phagocytes and increase fungal clearance (Oliveira et al., 2010). This immunomodulatory role was also observed in EVs released by *C. albicans*, in which the vesicles were able to stimulate innate and adaptive response and trigger an increase in fungal clearance (Vargas et al., 2015).

Of interest, most studies have characterized EVs in yeast, while the release and characterization of these structures in filamentous fungi, such as *Aspergillus fumigatus*, have been poorly explored. *A. fumigatus* is a filamentous, ubiquitous and saprophytic fungus of significant medical importance to humans (Latgé, 1999). To the best of our knowledge, the production, secretion and function of EVs in *A. fumigatus* has not been described. Considering the capacity of EVs to contribute to the pathogenesis of various fungal infection, we characterized and investigated the immune effects of *A. fumigatus* EVs. Our results demonstrated that the EVs are released by *A. fumigatus* and their production is affected by the time of growth. We also identified that EVs are able to stimulate phagocytes and improve the phagocytic capacity and fungal clearance by these cells.

## Materials and Methods

### Culture Conditions

The *A. fumigatus* A1163 strain was used to EVs isolation. This strain is derived from *A. fumigatus* CEA17, a strain converted in *pyrG*+ by the insert of *Aspergillus niger pyrG*+ gene. CEA17 strain is a uracil auxotrophic strain from *A. fumigatus* clinical isolate CEA10 (Fedorova et al., 2008). To standardize EVs production, 1 × 10^7^ conidia from *A. fumigatus* were inoculated in 50 mL of YG medium (0.5% w/v yeast extract powder; 2% w/v glucose; 0.1% v/v trace elements). Conidia were incubated for 24, 48, 72, 96, and 120 h under 120 rpm at 37°C. The mycelia was filtered in paper filter, then dried and weighed. After standardizing the time of culture, inoculums were made in 1 L of culture.

### EVs Isolation

**FIGURE 1:**
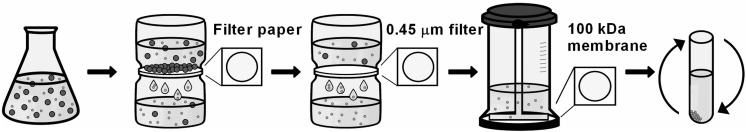
Experimental strategy to isolate EVs. 2 × 10^8^ conidia/L were inoculated in YG media for 48, 72, 96, and 120 h at 37°C. After mycelium filtration with paper filter, the supernatant was filtered in a 0.45 μm membrane to retain reminiscent cells. Then, the flow-through was concentrated using a 100 kDa membrane. The supernatant was concentrated at 100,000 *g* to isolate the vesicles pellet.

The isolation of EVs was performed according to Rodrigues and colleagues (Rodrigues et al., 2007), adapted. At each time point, the mycelium was separated from supernatant by filtration using paper filter. Supernatant was filtered using a 0.45 μm filter (Sartorius) and concentrated up to 25 mL Amicon ultra-concentration system (cutoff 100 KDa, Millipore). The concentrated supernatant was centrifuged at 100,000 *g* for 1 h at 4°C. The pellet of EVs was washed with phosphate-buffered saline 1X (PBS) and centrifuged in the same conditions. Pellet was resuspended in 260 μL PBS 1X, treated with Protease Inhibitor Cocktails 10X (Sigma) in 1:100 and stored at −80°C (Figure 1).

### Nanoparticle Tracking Analysis (NTA)

Quantity and distribution of EVs size were measured by NTA, using the Nanosight appliance (Malvern Instruments) and NTA 3.0 software. NTA is a technic of optical dispersion to determine the distribution of size, in a nanometer scale, of particles in a solution. The appliance allows a direct individual light dispersion visualization of particles illuminated by a laser beam (Rupert et al., 2017).

### Transmission Electronic Microscopy (TEM)

TEM was used to visualize the EVs from supernatant of *A. fumigatus* culture. Pellets obtained from six independent preparations were fixed with glutaraldehyde 2.5% v/v + 4% v/v formaldehyde in sodium cacodylate buffer 0.1 M; pH 7.2. Next, samples were washed in PBS and incubated for 60 min in 1% osmium, dehydrated in ethanol series, and embedded in Spurr’s resin. Ultrathin sections (70 nm) were obtained in Leica UC7 ultramicrotome and contrasted with 5% w/v uranyl acetate for 20 min and 0.5% w/v lead citrate for 5 min. Samples were observed in a JEOL 1200EX transmission electron microscope operating at 80 kV (Rodrigues et al., 2007; Albuquerque et al., 2008).

### Proteins and Ergosterol Quantification

Proteins were quantified using Bradford reagent (Bio-Rad) at 595 nm, using a standard curve (5–30 μg/μL) of Bovine Serum Albumin (BSA). Ergosterol was quantified by modifications of Vargas and colleagues protocol (Vargas et al., 2015). Briefly, speedvac dried EVs were resuspended in 50 μL methanol. It was added 450 μL of chloroform, homogenated and centrifuged at 18,000 *g* for 5 min. The supernatant dried in speedvac and resuspended in 200 μL of absolute ethanol. The quantification was done by colorimetry using a calibration curve (0.5–1.024 μg/mL) of ergosterol, at 282 nm.

### Animals

Experiments in mice were approved by the ethics committee (Comissão de Ética no Uso de Animais CEUA-255/2018) of Universidade Federal de Minas Gerais (UFMG). All experiments were performed in accordance with international guidelines and regulations. Ten to twelve weeks mice C57BL/6 were infected with 2 × 10^7^ conidia of *A. fumigatus* A1163, once a week, during 3 weeks. One week after the last infection, serum of animals was obtained and stored at −20°C. Animals were euthanized in order to remove tibia and fibula bones. These bones were washed with RPMI medium in order to obtain bone-marrow for neutrophils isolation. The content was centrifuged at 430 *g* for 10 min at 4°C. After that, red blood cells were lysed with ACK Lysis buffer and cells were centrifuged again in the same conditions. Histopaque 1077-1 (Sigma) (1:1) was used to separate the polymorphonuclear cells, prior to their centrifugation at 430 *g* for 30 min at 4°C.

### SDS-PAGE and Immunoblotting

Two μg of proteins of EVs were resuspended in sample buffer (Tris-HCl 0.5 M; Glycerol; SDS 10% w/v; β-mercaptoethanol; blue of bromofenol), and resolved in SDS-PAGE 12%. The total proteins profile was stained by silver.

For immunoblotting, 2 μg of proteins of EVs were resolved by SDS-PAGE and transferred to nitrocellulose membrane. After blocking with 5% w/v skimmed milk the membrane was incubated with serum from mice stimulated with *A. fumigatus* (1:10). After overnight incubation, anti-mouse IgG-HRP secondary antibody (Santa Cruz) was added for 1 h in room temperature. The membrane revealed with Luminata solution (Luminata Classico Western HRP Substrate, Millipore). Cropping, faint background, contrast and colors edition were done using Photos software from Windows 10.

### Proteomic Analysis by Liquid Chromatography-Tandem Mass Spectrometry

The lyophilized samples digested using ProteaseMAX stock (Promega) and 2 μg of trypsin. After digestion with trypsin was used μ-C18 ZipTip (Merck Millipore) for cleaning up peptide samples. After, the samples were dried in speedvac and used for the analysis.

An Easy-nLC 1200 system (Thermo Fisher Scientific Corp., Waltham, MA, United States) was coupled to an Orbitrap Fusion Lumos instrument equipped with a nanospray source (Thermo Fisher Scientific Corp., Waltham, MA, United States). Nano-LC solvents were water with 0.1% formic acid (A) and acetonitrile: water (80:20) with 0.1% formic acid (B) and the flow rate was 300 nL/min. Samples (3 μL) were injected onto a trapping column (Acclaim PepMap 0.075 mm, 2 cm, C18, 3 μm, 100 A; Thermo) in line with a Nano-LC column (Acclaim PepMap RSLC (0.075 mm, 15 cm, C18, 2 μm, 100 A; Thermo). The sample was loaded in the trap column and washed with 20 μL of solvent A at constant pressure (500 bar). After that, the sample was eluted to the column using a flow of 300 nL/min.

MS/MS analyses were conducted in the ESI^+^ mode. The instrument settings included the spray voltage at 1950 kV, capillary temperature at 300°C, and S-Lens RF level at 30%. A full-scan event was performed in profile mode over the mass range of *m/z* 400–1600 at a resolution of 120,000 followed by MS/MS analyses in a cycle time of 3 s. High-collision dissociation (HCD) with a normalized collision energy set at 30% was used for fragmentation. The resulting MS/MS fragment ions were detected in the mass range of *m/z* 100–2000 using the Orbitrap mass analyzer at a resolution of 30,000 using centroid mode. An AGC target of 5e^4^ and a maximum injection time of 54 ms were used.

*A. fumigatus* databank available at UniProt^1^ were loaded into MaxQuant (Tyanova et al., 2016) and used to identify the protein content on EVs. A combined list of proteins identified in all independent replicates (*n* = 3) were generated.

### Phagocytic and Clearance Ability of Immune Cells After Stimulation With Vesicles

Macrophages RAW 264.7 were cultivated in Dulbecco’s minimal essential medium (DMEM), supplemented with Fetal Bovine Serum 10% v/v (FBS). Cells were maintained at 37°C in a 5% CO_2_ atmosphere. 5 × 10^4^ macrophages were stimulated for 5 h with *A. fumigatus* EVs in a final amount of 0.1; 0.2; or 0.4 μg of proteins. After stimulation period, 5 × 10^5^ conidia of A1163 strain (Multiplicity of Infection – MOI 10:1) were added to cell culture. Content of wells was collected after 6 h of stimulus. Macrophages were washed with 1X PBS to remove the non-phagocyted conidia, lysed with sterile water, and conidia were plated in YAG medium to quantify the colony forming units (CFU).

To evaluate phagocytosis, 5 × 10^4^ macrophages were stimulated for 5 h with EVs in a final amount of 0.4 μg of proteins. 5 × 10^5^ conidia of A1163 strain (MOI 10:1) were added to cell culture. After 4 and 6 h of stimulus, supernatant was collected, cells were stained with Quick Panoptic (Laborclin).

The capacity of fungal clearance by cells stimulated with EVs was also evaluated in neutrophils. 5 × 10^5^ neutrophils (BMDN) were stimulated for 3 h with EVs in a final amount of 0.4 μg of proteins, at 37°C in 5% CO_2_ atmosphere. 5 × 10^5^ conidia (MOI 1:1) were added to culture of cells. After 3 h of challenge, content of wells was collected, neutrophils were lysed with sterile water and the reminiscent conidia were plated in YAG medium to quantify the CFU.

To evaluate phagocytosis, 5 × 10^5^ neutrophils were stimulated for 3 h with EVs (0.4 μg of proteins). 2.5 × 10^6^ conidia of A1163 strain (MOI 5:1) were added to neutrophil culture. After 3 h, supernatant was collected and submitted to a citospin for 5 min at 35 *g*, the slides were stained with Quick Panoptic (Laborclin). Non-stimulated cells, cells stimulated only with EVs and cells challenged only with the fungus were used as control.

### Confocal Microscopy

5 × 10^5^ macrophages RAW 264.7 were plated in a 24 wells plate. The cells were incubated with CellMask Green (Thermo Fisher Scientific) in order to stain them, at 37°C in a 5% CO_2_ atmosphere, for 10 min. *A. fumigatus* EVs were treated with 3 μM of DilC_18_ (Thermo Fisher Scientific) for 1 h. EVs were washed one time with PBS 1X, resuspended in DMEM medium and co-incubated with macrophages for 30 min (Nicola et al., 2009; Oliveira et al., 2010). After this period, well was washed 3 times with DMEM medium and the slice submitted to confocal microscopy (4X zoom, increase of 20X).

### Cytokines Measurement

Supernatant of cells stimulated with EVs and challenged with *A. fumigatus* were obtained and the levels of pro-inflammatory cytokines and chemokine TNF-α, IL-1β, IFN-γ, and CCL2, and the modulatory cytokine IL-10 were evaluated, according to manufacturer instructions (R&D Systems).

### Statistical Analysis

Statistical differences among experimental groups were determined by one way analysis of variance (One Way-ANOVA), followed by Tukey post-test. Results involving two experimental groups were analyzed by t Student test. All data was considered statistically significant if *p* < 0.05. Statistical analysis were realized using the GraphPad Prism 6 software.

## Results

### *A. fumigatus* Produce EVs During Growth

**FIGURE 2:**
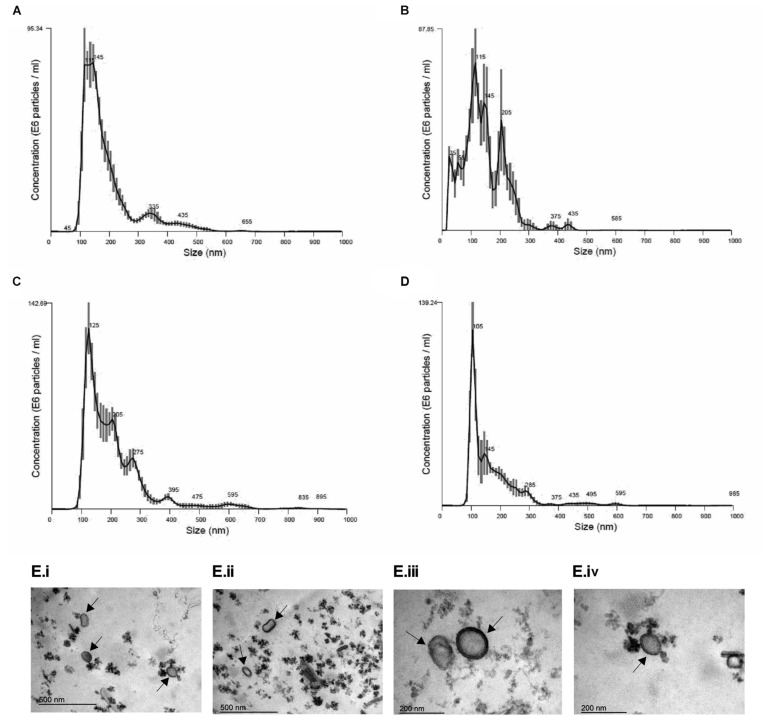
Morphological and dimensional aspects of *A. fumigatus* EVs. Dimensional analysis of EVs in 48 h **(A)**, 72 h **(B)**, 96 h **(C),** and 120 h **(D)** of culture. EVs were isolated and observed by Nanoparticle Tracking Analysis – Nanosight (Malvern). E6 particles means 10^6^ particles. Transmission Electron Microscopy (TEM) of EVs isolated by ultracentrifugation of 48 h culture supernatants from *A. fumigatus* **(E)**. Scale bar 500 nm **(i,ii)** and 200 nm **(iii,iv)**. Arrows indicate EVs.

In order to establish conditions of growth and production of EVs, we analyzed different culture time points (24, 48, 72, 96, and 120 h at 37°C). It was observed that fungal mass increased exponentially until 72 h and the growth speed decreased after that (Supplementary Figure S1), suggesting this time point was the beginning of the stationary phase. Based on the *A. fumigatus* growth curve, we analyzed the size and yield of EVs production. We observed that the yield of EVs production was similar in all time points (Table 1). Besides that, *A. fumigatus* produced a majority amount of EVs with average size between 100 and 200 nm and a minor population of EVs varying from 300 to 595 nm (Figures 2A–D). Further experiments were conducted using fungi collected at 48 h of culture, as this was optimal in terms of growth, time of incubation and yield. Using these 48 h cultures, Transmission Electronic Microscopy (TEM) analysis revealed the presence of spherical structures displaying electrodense bilayers, characteristics of EVs. Quantification showed that these isolated EVs had the same average diameter as demonstrated by NTA (Figure 2E).

**TABLE 1:** Concentration of EVs released by *A. fumigatus* in different periods of culture.

**NTA analysis**	

**Period of culture (h)**	**Concentration (particles/mL)**
48	8.56 × 10^9^ ± 3.76 × 10^8^
72	8.91 × 10^9^ ± 4.91 × 10^8^
96	1.33 × 10^10^ ± 3.51 × 10^8^
120	6.67 × 10^9^ ± 3.93 × 10^8^

### *A. fumigatus* EVs Composition

**FIGURE 3:**
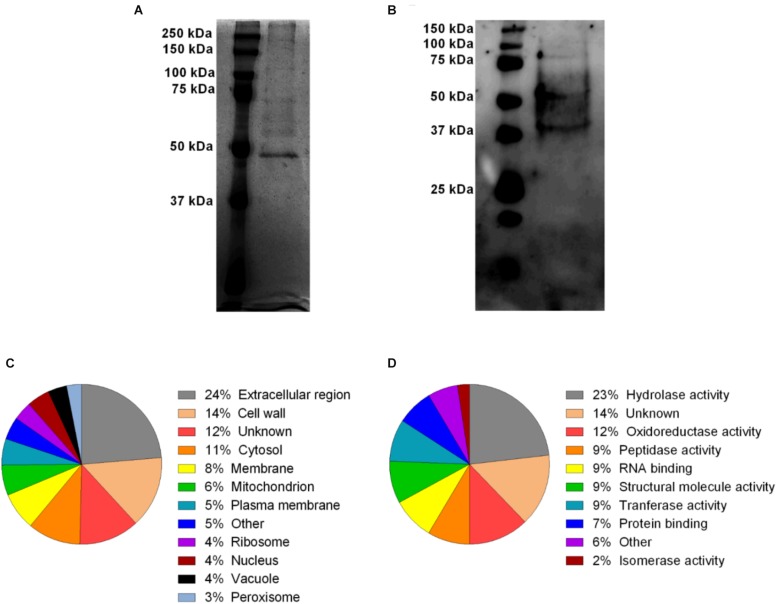
*A. fumigatus* EVs protein diversity and Gene Ontology categorization of MS identified proteins. The content of proteins (2 μg) in the vesicles was visualized by silver staining **(A)**. Some of these proteins were reactive in the presence of total anti-serum from previously infected mice **(B)**. Proteins were identified by mass spectrometry and classified according to their subcellular predicted localization **(C)** and molecular predicted function **(D)**.

In order to investigate the profile of protein content in *A. fumigatus* EVs, a silver stained SDS-PAGE was performed. Figure 3A shows the electrophoretic resolved profile of protein content in the EVs demonstrating at least 9 major bands of proteins varying from 50 to 250 KDa (Supplementary Figure S2). These proteins were transferred to nitrocellulose membrane and incubated with total serum obtained from mice previously infected with *A. fumigatus*. Serum reactive proteins of approximately 37–150 kDa were found in *A. fumigatus* EVs (Figure 3B and Supplementary Figure S3).

Considering that the protein profile in *A. fumigatus* EVs is unknown, the protein composition was identified using proteomics. According to our analysis, 60 proteins were identified (Table 2). The cellular composition and the molecular and biological functions proposed for these proteins are shown in Supplementary Table S1. Using the UniProt^2^ and AspGD^3^ databases, proteins were classified according to their predicted cellular localization and molecular putative functions in the fungal cells (Figures 3C,D), and included hydrolases, oxydoreductase, peptidases, tranferases, RNA/carbohydrate/protein binding, structural activity, isomerases and phosphatases. The proteins were also classified according to cellular localization. The identified proteins were associated to the extracellular region (24%), cell wall (14%), cytosol (11%), membrane (8%), mitochondrion (6%), plasma membrane (5%), ribosome (4%), nucleus (4%), vacuole (4%) and peroxisome (3%) (Figure 3C). The proteins were also classified according to their biological function. The majority of vesicular proteins classified were associated to carbohydrate metabolic processes and response to cellular stress, and also to proteins associated to pathogenesis (Supplementary Table S1). The presence of proteins with antigenic potential, in association with pathogenesis and host response stimulation in EVs proteome, led us to investigate if the *A. fumigatus* EVs were able to stimulate macrophages and neutrophils, as competent phagocytes.

**TABLE 2:** List of protein groups identified by proteomic analysis of extracellular vesicles from *A. fumigatus.*

**Protein group number**	***A. fumigatus* genome database acession number**	**Protein name**
1	AFUB_063700	Glutamate dehydrogenase
2	AFUB_016770	Uncharacterized protein
3	AFUB_063890	Ecm33
4	AFUB_096050	Allergen Asp F3 (Peroxiredoxin family protein)
5	AFUB_037910	Ubiquitin (UbiC), putative
6	AFUB_015530	Extracellular cell wall glucanase Crf1/allergen Asp F9
7	AFUB_066130	Aminopeptidase
8	AFUB_094680	FAD/FMN-containing isoamyl alcohol oxidase MreA
9	AFUB_094730	IgE-binding protein, putative
10	AFUB_097210	Carboxypeptidase
11	AFUB_095500	GPI anchored protein, putative
12	AFUB_005920	Glycogenin
13	AFUB_002680	Uncharacterized protein
14	AFUB_099560	Tripeptidyl-peptidase (TppA), putative
15	AFUB_004489	FG-GAP repeat protein, putative
16	AFUB_045170	Cell wall protein phiA
17	AFUB_048140	Extracellular phytase, putative
18	AFUB_020900	Allergen Asp F4
19	AFUB_022370	1,3-beta-glucanosyltransferase gel4
20	AFUB_052010	Nucleoside diphosphate kinase
21	AFUB_052060	Thioredoxin reductase, putative
22	AFUB_052270	Class III chitinase ChiA1
23	AFUB_050860	Major allergen Asp F1
24	AFUB_052690	Molecular chaperone Mod-E/Hsp90
25	AFUB_085650	Endo-chitosanase
26	AFUB_046050	Alpha,alpha-trehalose glucohydrolase TreA/Ath1
27	AFUB_048180	Probable glucan endo-1,3-beta-glucosidase eglC
28	AFUB_047510	Extracellular conserved serine-rich protein
29	AFUB_047560	FAD-dependent oxygenase, putative
30	AFUB_037350	Phosphoglucomutase PgmA
31	AFUB_050510	BYS1 domain protein, putative
32	AFUB_040810	Aspartyl aminopeptidase
33	AFUB_010890	1,3-beta-glucanosyltransferase Bgt1
34	AFUB_023440	60S ribosomal protein L18
35	AFUB_018250	1,3-beta-glucanosyltransferase gel1
36	AFUB_009540	Adenosylhomocysteinase
37	AFUB_066060	GPI anchored cell wall protein, putative
38	AFUB_087520	Isoamyl alcohol oxidase, putative
39	AFUB_006000	40S ribosomal protein S3, putative
40	AFUB_004410	Ubiquitin UbiA, putative
41	AFUB_079620	Uncharacterized protein
42	AFUB_050490	Glyceraldehyde-3-phosphate dehydrogenase
43	AFUB_023550	Probable Xaa-Pro aminopeptidase pepP
44	AFUB_005160	Probable NAD(P)H-dependent D-xylose reductase xyl1
45	AFUB_036480	Putative UDP-galactopyranose mutase
46	AFUB_034560	Uncharacterized protein
47	AFUB_093550	Actin Act1
48	AFUB_021670	ER Hsp70 chaperone BiP, putative
49	AFUB_006770	Elongation factor 1-alpha
50	AFUB_056780	Superoxide dismutase [Cu-Zn]
51	AFUB_007770	Molecular chaperone Hsp70
52	AFUB_009760	Phosphoglycerate kinase
53	AFUB_036860	60S ribosomal protein L22, putative
54	AFUB_025910	60S acidic ribosomal protein P2/allergen Asp F 8
55	AFUB_017890	Purine nucleoside permease, putative
56	AFUB_000660	CFEM domain protein
57	AFUB_089500	Uncharacterized protein
58	AFUB_070900	Uncharacterized protein
59	AFUB_024920	Dipeptidyl-peptidase 5
60	AFUB_001190	Ribosomal protein S13p/S18e

Ergosterol is the main lipid in fungal membranes and its content is also important to maintain plasma membrane integrity in EVs. Results demonstrated that in 1.5 × 10^10^ *A. fumigatus* EVs particles there were 13.5 μg of ergosterol.

### *A. fumigatus* EVs Are Recognized by Macrophages and Promote an Increase of Fungicide Capacity and Production of Inflammatory Mediators

**FIGURE 4:**
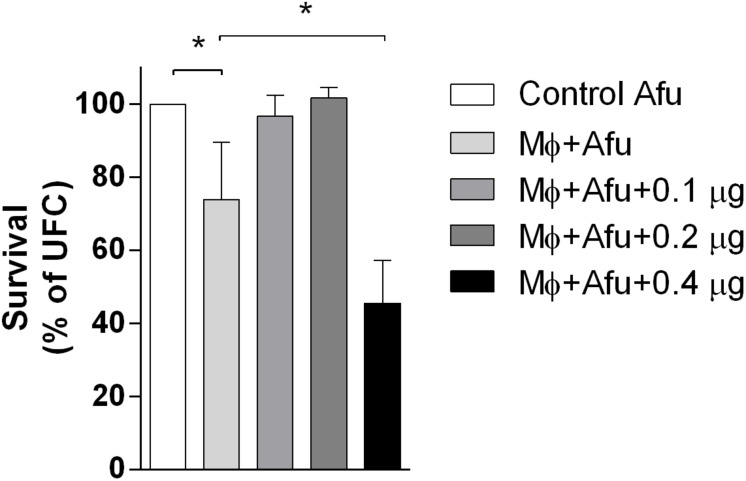
*A. fumigatus* killing by macrophages. 5 × 10^4^ RAW 264.7 macrophages were stimulated with 0.1, 0.2, and 0.4 μg of EVs (measured as total protein content) for 5 h. After that, the macrophages were challenged with 5 × 10^5^ (MOI 10:1) conidia of *A. fumigatus* for 6 h. The amount of live conidia was quantified in YG media as unit forming colony (UFC). Data are representative of three experiments. ^∗^ Represents statistically differences between the indicated groups (*p* < 0.05) as determined by One Way-ANOVA.

EVs are known to modulate host responses and function as virulence factors. In order to identify the effects of *A. fumigatus* EVs in phagocytes, macrophages were stimulated with different amounts of EVs and challenged with *A. fumigatus*. Results demonstrated that 0.1 and 0.2 μg of EVs (measured as total protein content) were not able to induce *A. fumigatus* clearance by macrophages (Figure 4). However, 0.4 μg of EVs were very effective in stimulating the clearance of conidia by macrophages, leading to more than 50% killing of conidia after challenge. When compared to the positive control, the stimulation of phagocytes with 0.4 μg of EVs was able to double the killing effector functions of macrophages (Figure 4).

**FIGURE 5:**
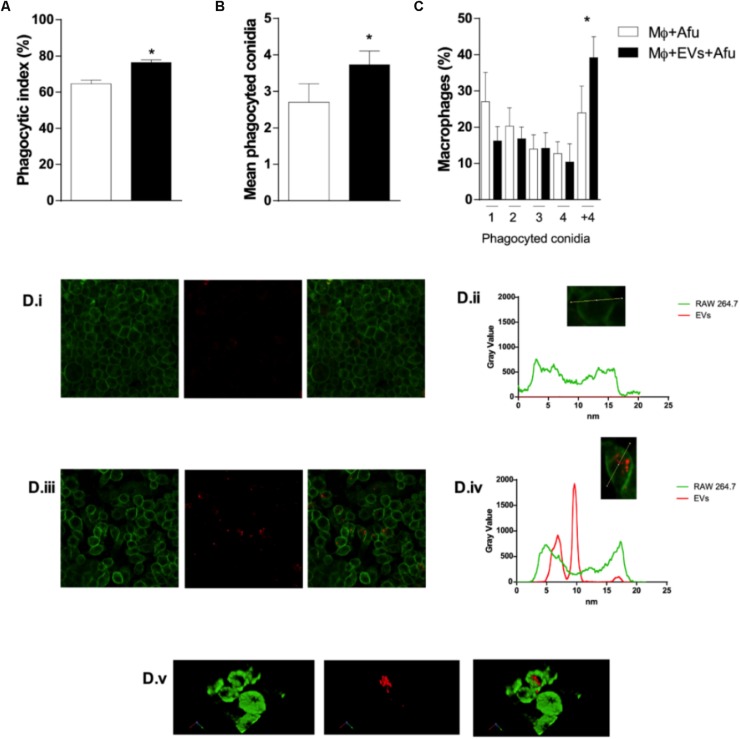
EVs are able to modulate *A. fumigatus* phagocytosis. 5 × 10^4^ macrophages were stimulated with EVs for 5 h. Then, the macrophages were challenged with 5 × 10^5^ (MOI 10:1) conidia of *A. fumigatus* for 4 h and the phagocytosis was evaluated **(A)**, the average number of phagocyted conidia by cells **(B)**, and the mean number of conidia phagocyted per macrophage **(C)**. DiIC_18_ (red)-stained vesicles were incubated with macrophages **(D)**. The plasma membrane was stained with CellMask (green). Control microscopy shown macrophages stained by CellMask without vesicles **(i)**; densitometry of a single cell (macrophage) stained by Cellmask **(ii)**; microscopy shown macrophages stained by CellMask incubated with DiLC_18_ stained vesicles **(iii)**; densitometry of a single cell (macrophage) stained by CellMask showing EVs in the cytoplasm **(iv)**. Co-localization of macrophages and vesicles in merged images **(v)**. (3D reconstruction). Data are representative of three experiments. ^∗^Represents statistically differences between the indicated groups (*p* < 0.05) as determined by One Way-ANOVA and two-sample independent *t*-tests.

In order to verify whether the mechanisms of fungal recognition and internalization by macrophages were affected by EVs, the phagocytic capacity of macrophages previously stimulated with *A. fumigatus* EVs was evaluated. After 5 h incubation, results demonstrate that EVs were able to increase phagocytosis by approximately 12% in comparison to macrophages not exposed to EVs (Figure 5A). In addition, complementary analysis showed that the number of phagocyted conidia in the EVs stimulated macrophages was higher than in the control group (Figure 5B). Macrophages from both groups (non-EVs or EVs stimulated) demonstrated an equivalent phagocytic capacity, in terms of internalizing 1, 2, 3, or 4 conidia per macrophage. On the other hand, the presence of EVs was able to induce macrophages capacity to internalize conidia as seen by an increase of almost 50% in the number of macrophages with more than 4 fungal cells inside cells (Figure 5C).

The phagocytosis of EVs by macrophages was observed under confocal microscopy. Images show that there were vesicles outside and inside macrophages. 3D reconstitution of images demonstrated that EVs were in the cytoplasm of macrophages, confirming their internalization (Figures 5Di–v and Supplementary Movie S1).

**FIGURE 6:**
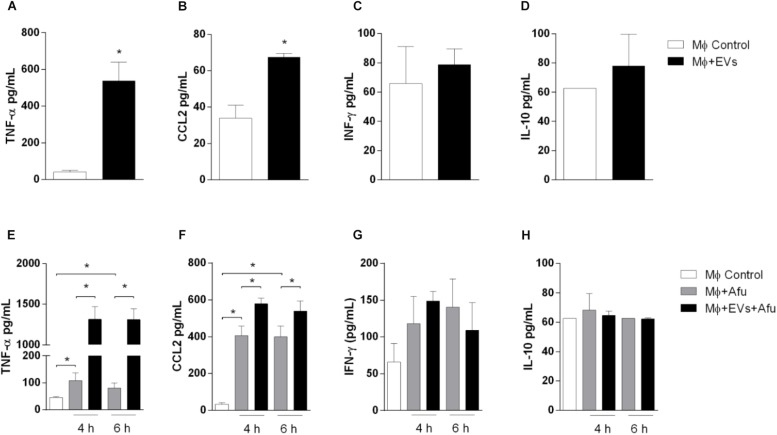
EVs are able to stimulate cytokines and chemokines production by macrophages. 5 × 10^4^ macrophages were stimulated with 0.4 μg of *A. fumigatus* EVs. Secretion of inflammatory mediators TNF-α **(A)**, CCL2 **(B)**, IFN-γ **(C)**, and IL-10 **(D)** were quantified. Cells pre-incubated with EVs were challenged with 5 × 10^5^ (MOI 10:1) conidia of *A. fumigatus* for 4 and 6 h. Production of inflammatory mediators TNF-α **(E)**, CCL2 **(F)**, IFN-γ **(G)**, and IL-10 **(H)**, during *in vitro* challenge with *A. fumigatus*, were quantified. Data are representative of three experiments. ^∗^Represents statistically differences between the indicated groups (*p* < 0.05) as determined by One Way-ANOVA and two-sample independent *t*-tests.

Considering that EVs are able to increase the uptake of the fungus by macrophages, levels of inflammatory mediators produced by EVs stimulation were evaluated. After 5 h stimulation, EVs were able to induce significant secretion of pro-inflammatory mediators TNF-α (approximately 10 times) and a twofold change in CCL2 levels compared to non-stimulated cells (Figures 6A,B). Results demonstrated no changes in IFN-γ levels and IL-10, after EVs stimulation (Figures 6C,D).

We analyzed the production of cytokines during *in vitro A. fumigatus* infection. Results demonstrate that EVs stimulation prior to fungal challenge is responsible for an increase of approximately 15 times in the production of TNF-α (Figure 6E). The levels of the chemokine CCL2 were also increased, around 30%, when compared to non-EVs control group (Figure 6F). No differences were observed in IFN-γ and IL-10 levels (Figures 6G,H).

### EVs Promote an Increase in Fungicide Capacity by Neutrophils and Enhance Cytokine Production After *A. fumigatus* Challenge

**FIGURE 7:**
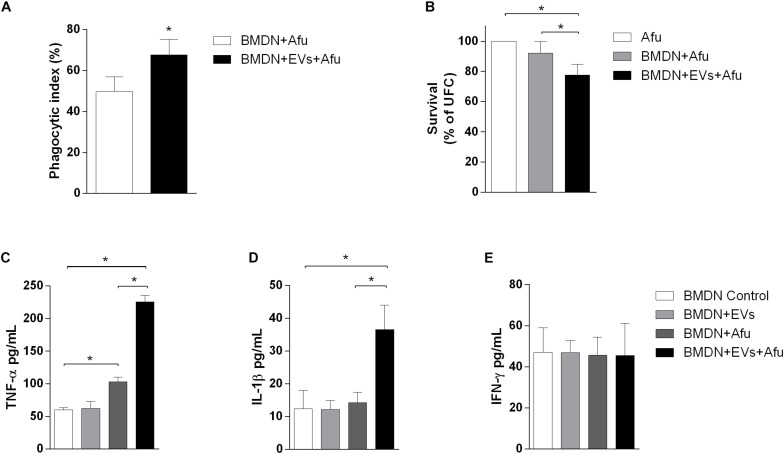
Fungicide capacity and cytokines production by neutrophils. 5 × 10^5^ bone marrow-derived neutrophils were challenged with 2.5 × 10^6^ (MOI 5:1) **(A)** or 5 × 10^5^ (MOI 1:1) conidia **(B)**, and the phagocytosis **(A)** and fungal killing **(B)** were evaluated after 3 h of incubation. Production of TNF-α **(C)**, IL-1β **(D)**, and IFN-γ **(E)** was evaluated in BMDN stimulated with EVs and challenged with *A. fumigatus.* Data are representative of three experiments. ^∗^Represents statistically differences between the indicated groups (*p* < 0.05) as determined by One Way-ANOVA and two-sample independent *t*-tests.

Neutrophils are recruited in response to *A. fumigatus* challenge (Dagenais and Keller, 2009; Muller, 2013). We investigated the capacity of EVs to stimulate bone marrow-derived neutrophils (BMDN) and to improve their fungicide capacity. After 3 h of EVs stimulation, there was a pattern of response similar to that of macrophages. EVs stimulation could enhance phagocytic capacity by approximately 17% when compared to the control non-EVs group (Figure 7A). This effect was accompanied by induction of fungal clearance by BMDN (Figure 7B). Together, these results show that the sensitization of phagocytes with *A. fumigatus* EVs was able to prime these cells and increase their phagocytic capacity, hence culminating in higher fungal clearance.

Considering that the EVs were able to induce the production of inflammatory mediators by macrophages, we also evaluated the capacity of these structures to stimulate cytokine production in BMDN. After 3 h of stimulus, EVs were not able to induce TNF-α, IL-1β, and IFN-γ production (Figures 7C–E, respectively). On the other hand, results demonstrated that stimulation with EVs induced an increase of TNF-α and IL-1β production after fungal challenge. Levels of IFN-γ did not change after challenge (Figures 7C–E).

## Discussion

In this work, we demonstrated for the first time the release of EVs by the pathogenic filamentous fungus, *A. fumigatus*. Considering that the liberation of EVs can happen in both the saprophytic phase or during the colonization of mammal hosts, different *in vitro* growth conditions could interfere with EVs production. In this sense, we analyzed several culture time points to determine the ideal condition to isolate the EVs released by *A. fumigatus*. The exponential phase of fungal growth is the period of highest metabolic activity of cells (Brown et al., 2015). According to our data and the literature, this time point for *A. fumigatus* comprises up to 72 h, when the stationary phase begins (Meletiadis et al., 2001; Reeves et al., 2004). Considering the growth of the fungus and that the production of EVs was similar at 48 and 72 h, 48 h of incubation was chosen as the optimal point to carry out the functional characterizations of *A. fumigatus* EVs.

In all *A. fumigatus* growing conditions two main populations of vesicles were identified, one major population constituted by small vesicles with size between 100 and 200 nm diameter and a minor population of vesicles up to 595 nm. Variations in the diameters of isolated EVs may indicate that different pathways may be important in their biogenesis. Our findings are consistent with the diameter of EVs observed in other fungi such as in *H. capsulatum*, *C. neoformans*, and *P. brasiliensis* (Rodrigues et al., 2007; Albuquerque et al., 2008; Vallejo et al., 2011; Baltazar et al., 2016). Other *A. fumigatus* strains probably release EVs with differences in size and consequently in composition, once it was already demonstrated that EVs released by different *C. neoformans* strains showed different protein composition. In *C. albicans* the EVs population can vary from 50 to 100 nm up to larger populations from 450 to 850 nm or 350 to 450 nm depending on the strain, and also showed differences in their protein content, demonstrating that fungal EVs are considered heterogeneous among species (Albuquerque et al., 2008; Vargas et al., 2015). The differences in EVs using other *A. fumigatus* strains will be investigated in future studies.

Secretion of EVs is considered an important vehicle of molecule transportation to the extracellular environment. The importance of EVs as a way of transportation is sustained by the fact that a great number of proteins have been identified in pathogenic fungi EVs. A variety of functions have been associated with these identified proteins, as biofilm formation, cell wall organization and remodeling, carbohydrates, lipids and protein metabolism, cell response to drugs, heat shock proteins, transport and vesicular fusion and antioxidants (Albuquerque et al., 2008; Rodrigues et al., 2008; Vargas et al., 2015; Zarnowski et al., 2018). In this work it was demonstrated *that A. fumigatus* EVs proteins were recognized by total serum antisera from previously *A. fumigatus* infected mice, suggesting that proteins of *A. fumigatus* transported by EVs are antigenic and sensitize host immune system. Similar findings have been reported for other fungal species, such as *H. capsulatum*, *C. neoformans*, *P. brasiliensis*, and *C. albicans* (Albuquerque et al., 2008; Rodrigues et al., 2008; Vallejo et al., 2011; Vargas et al., 2015).

The characterization of the proteins inside EVs identified proteins involved in metabolic processes, filamentous growth, sporulation, cell cycle and transport. Most of the proteins were classified as hydrolases. The presence of proteins that are able to hydrolyze the components of cell wall is interesting, once they can promote the remodeling of cell wall allowing the secretion of EVs (Albuquerque et al., 2008; Brown et al., 2015). Among proteins involved in cell wall remodeling in *A. fumigatus*, we identified the presence of glucanosyltransferases (Gel1, Gel4, and Bgt1), Ecm33 and EglC that participate in the elongation of cell wall glucan chain leading to maintenance and resistance of cell wall, suggesting that these proteins in the EVs participate of mechanisms of fungal growth (Chabane et al., 2006; Gastebois et al., 2010; Zhao et al., 2013; Champer et al., 2016).

Many proteins were predicted to localize in the intracellular or extracellular space, suggesting that these vesicles are serving as a mechanism of transport for proteins beyond cells. Some identified proteins, as nucleoside diphosphate kinase and superoxide dismutase, have an important role in *A. fumigatus* polarization growth and in the resistance to high temperatures (Momany et al., 1999; Lin et al., 2003; Lambou et al., 2010; Dinamarco et al., 2012). These proteins are also involved in the pathogenesis and host response activation. Nucleoside diphosphate kinase plays a key role in resistance to oxidative stress. It was demonstrated in *Neurospora crassa* that a knockout mutant for this protein showed hypersensitivity to oxidative and thermal stress (Yoshida et al., 2006). Superoxide dismutase detoxifies superoxide reactive anions and contributes to oxidative burst inhibition. *A. fumigatus* SODs knockout mutants showed sensitivity to higher temperatures and ROS donors and their clearance by *in vitro* macrophages were higher than the wild type (Holdom et al., 2000; Lambou et al., 2010). In the same way, the thioredoxin reductase is able to inhibit the respiratory burst in neutrophils by breaking the NADPH oxidase, facilitating the fungus dissemination (Tsunawaki et al., 2004; Shi et al., 2012).

Chaperones are involved in morphogenesis, stress response (temperature and pH), osmolarity and antifungal resistance. This family of proteins were also identified in EVs of others fungi, such as *C. neoformans*, *H. capsulatum*, and *C. albicans* (Albuquerque et al., 2008; Rodrigues et al., 2008; Vargas et al., 2015) and are related to virulence and resistance to antifungals (Cordeiro et al., 2016; Cleare et al., 2017; O’Meara et al., 2017). HSP90 is an essential component of cytoplasmic chaperone network HSP70-HSP90 responsible for protein folding. Besides that, HSP90-calcineurin pathway have a crucial role in the antifungal resistance (Soriani et al., 2008; O’Meara and Cowen, 2014; Tiwari et al., 2015).

Other components of significant relevance to *A. fumigatus* virulence are proteins known to possess significant allergenic properties in the host. In the EVs of *A. fumigatus* the allergens Asp f-1, Asp f-3, Asp f-4, Asp f-8, and Asp f-9 were identified. The ribotoxin Asp f-1 is able to reach the cytosol of mammalian host cells and inactivate the ribosomes inhibiting the proteins synthesis (Olmo et al., 2001; Lacadena et al., 2007). Asp f-3 is a peroxiredoxin able to bind IgE and inactivate ROS (Ramachandran et al., 2002; Hillmann et al., 2016). Asp f-4 is also one of the main antigens of *A. fumigatus*, with unknown function, and it is used as a marker for diagnosis of allergic bronchopulmonary aspergillosis (ABPA) (Kurup et al., 2000). Together, these data suggest that the milieu of proteins in *A. fumigatus* EVs have a role in invasion, resistance and host fungus growth, demonstrating that EVs secretion may be related to virulence of *A. fumigatus*.

Studies with EVs of pathogenic microorganisms demonstrate that EVs are able to interfere with a proper immune response. *A. fumigatus* EVs increased fungal killing by phagocytes and this property is in accordance with results described for EVs derived from *C. neoformans* (Oliveira et al., 2010), *Trichophyton interdigitale* (Bitencourt et al., 2018), *S. aureus* (Choi et al., 2015), and *C. albicans* in a *Galleria mellonella* larva model of infection (Vargas et al., 2015). Together, these and our study show that EVs are immunologically active and have a potential to interfere with the course of infection, in general causing higher clearance of the pathogen after stimulation with the vesicles. These findings were unexpected as the fungus would be spending a considerable amount of energy producing EVs with proteins that do no favors its relation with host cells. However, these same protein may be associated with fungal growth and communication and enhancement of immune responses could be an unwanted effect (to the fungus) of secreted EVs. Further studies, however, are necessary to fully characterize the role of these EVs for fungal physiology and during *in vivo* infection, where multiple interactions between various cell types may occur.

The immunomodulatory components of EVs from pathogens indicate that the molecules associated to EVs can promote survival and dissemination of these organisms. On the other hand, they can also stimulate host immune response for pathogen clearance. The exact role of EVs derived from pathogens in the host-pathogen interactions probably depends on the development stage of the pathogens, environmental conditions and/or specific tissue (Schorey et al., 2015; Kuipers et al., 2018). In our work, we identified that the components present in *A. fumigatus* EVs have potential in stimulating an *in vitro* pro-inflammatory response, and increasing the capacity of clearance of the fungus.

Exposure of phagocytic cells to *A. fumigatus* was able to induce inflammatory mediators production, and interestingly, cells previously stimulated with EVs showed an additive effect in production of TNF-α, IL-1β, and CCL2. In *A. fumigatus* infections, TNF-α is a critical component of the innate immune response, as its signaling deficiency is able to result in a decrease of neutrophil influx and increase in mortality of infected animals. The administration of a TNF-α agonist, prior to infection with *A. fumigatus*, is able to increase the survival of mice (Mehrad et al., 1999). CCL2 is one of the main chemoattractant molecules regulating innate immunity, and it is positively regulated during *in vitro* challenge by *A. fumigatus*. The administration of anti-CCL2 serum in mice was able to decrease the clearance of conidia and increase the hyper-reactivity into the airways of mice infected with *A. fumigatus* (Blease et al., 2001; Loeffler et al., 2009).

## Conclusion

In conclusion, we demonstrated that *A. fumigatus* produce EVs that are rich in a range of bioactive proteins. EVs production is affected by different environmental conditions, which suggests that these structures can have an important function during growth. EVs can be recognized by phagocytes and stimulate phagocytosis and production of pro-inflammatory mediators and impact on fungal clearance.

## Data Availability

The raw data supporting the conclusions of this manuscript will be made available by the authors, without undue reservation, to any qualified researcher.

## Ethics Statement

Animal Subjects: The animal study was reviewed and approved by Comissão de Ética no Uso de Animais (CEUA), 255/2018, of Universidade Federal de Minas Gerais (UFMG).

## Author Contributions

FS conceived the study. JS and FS designed the experiments and wrote the manuscript. JS, LB, VC, LG-E, AO, WD, MR, KM, and IM performed the experiments. JS, LB, VC, LG-E, AO, WD, KM, IM, and FS interpreted the results and analyzed the data. LB, VC, LG-E, AO, WD, KM, IM, DAS, FF, DGS, MT, and FS contributed reagents, materials, and analysis tools. All authors read and approved the final manuscript.

## Conflict of Interest Statement

The authors declare that the research was conducted in the absence of any commercial or financial relationships that could be construed as a potential conflict of interest.

## Footnotes

1 https://www.uniprot.org

2 https://www.uniprot.org/

3 http://www.aspgd.org/
