# Corrigendum: Characterization of *Aspergillus fumigatus* Extracellular Vesicles and Their Effects on Macrophages and Neutrophils Functions

**DOI:** 10.3389/fmicb.2019.02334

**Published:** 2019-10-11

**Authors:** Jéssica Amanda Marques Souza, Ludmila de Matos Baltazar, Virgínia Mendes Carregal, Ludmila Gouveia-Eufrasio, André Gustavo de Oliveira, Wendell Girard Dias, Marina Campos Rocha, Kildare Rocha de Miranda, Iran Malavazi, Daniel de Assis Santos, Frédéric Jean Georges Frézard, Daniele da Glória de Souza, Mauro Martins Teixeira, Frederico Marianetti Soriani

**Affiliations:** ^1^Centro de Pesquisa e Desenvolvimento de Fármacos, Departamento de Genética, Ecologia e Evolução, Instituto de Ciências Biológicas, Universidade Federal de Minas Gerais, Belo Horizonte, Brazil; ^2^Laboratório de Interação Microrganismo-Hospedeiro, Departamento de Microbiologia, Instituto de Ciências Biológicas, Universidade Federal de Minas Gerais, Belo Horizonte, Brazil; ^3^Laboratório de Biofísica e Sistemas Nanoestruturados, Departamento de Fisiologia e Biofísica, Universidade Federal de Minas Gerais, Belo Horizonte, Brazil; ^4^Laboratório de Micologia, Departamento de Microbiologia, Universidade Federal de Minas Gerais, Belo Horizonte, Brazil; ^5^Lab Circuitos Fisiológicos, Departamento de Fisiologia e Biofísica, Universidade Federal de Minas Gerais, Belo Horizonte, Brazil; ^6^Plataforma de Microscopia Eletrônica Rudolf Barth, Fundação Oswaldo Cruz, Rio de Janeiro, Brazil; ^7^Centro de Ciências Biológicas e da Saúde, Departamento de Genética e Evolução, Universidade Federal de São Carlos, São Carlos, Brazil; ^8^Laboratório de Ultraestrutura Celular Hertha Meyer, Programa de Biologia Celular e Parasitologia, Instituto de Biofísica Carlos Chagas Filho, Universidade Federal do Rio de Janeiro, Rio de Janeiro, Brazil; ^9^Centro de Pesquisa e Desenvolvimento de Fármacos, Departamento de Bioquímica e Imunologia, Instituto de Ciências Biológicas, Universidade Federal de Minas Gerais, Belo Horizonte, Brazil

**Keywords:** extracellular vesicles, filamentous fungus, *Aspergillus fumigatus*, host-pathogen interactions, macrophages, neutrophils

Due to an oversight Marina Campos Rocha was not included as an author in the published article. The corrected Author Contributions Statement appears below:

## Author Contributions

FS conceived the study. JS and FS designed the experiments and wrote the manuscript. JS, LB, VC, LG-E, AO, WD, MR, KM, and IM performed the experiments. JS, LB, VC, LG-E, AO, WD, KM, IM, and FS interpreted the results and analyzed the data. LB, VC, LG-E, AO, WD, KM, IM, DAS, FF, DGS, MT, and FS contributed reagents, materials, and analysis tools. All authors read and approved the final manuscript.

The authors apologize for this error and state that this does not change the scientific conclusions of the article in any way. The original article has been updated.

