# Supplementary material for: Characterization of Aspergillus fumigatus Extracellular Vesicles and Their Effects on Macrophages and Neutrophils Functions
Source: Front Microbiol. 2019 Sep 4;10:2008. doi: 10.3389/fmicb.2019.02008 (PMC6738167; doi:10.3389/fmicb.2019.02008)
Supplement: Supplementary file 1 [file Data_Sheet_1.pdf]

Supplementary Material

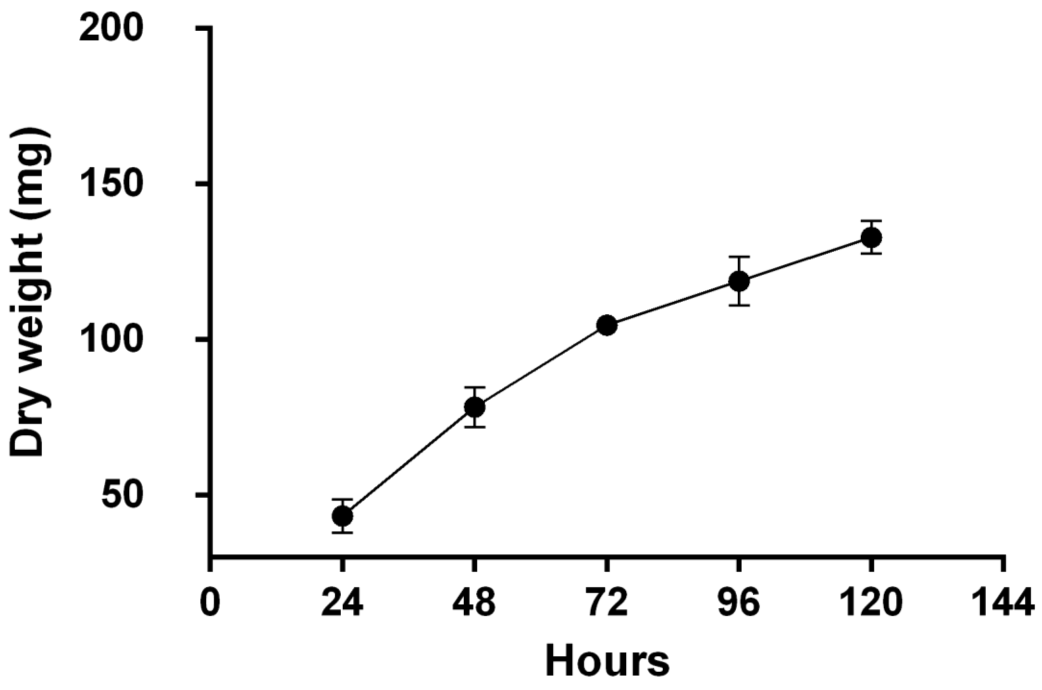

Figure S1. Growth curve of *A. fumigatus* culture. 2x10^8 conidia/L of *A. fumigatus* A1163 were inoculated in 50 mL of complete YG at 37°C and dried fungal mass was analyzed after 24, 48, 72, 96 and 120 hours.

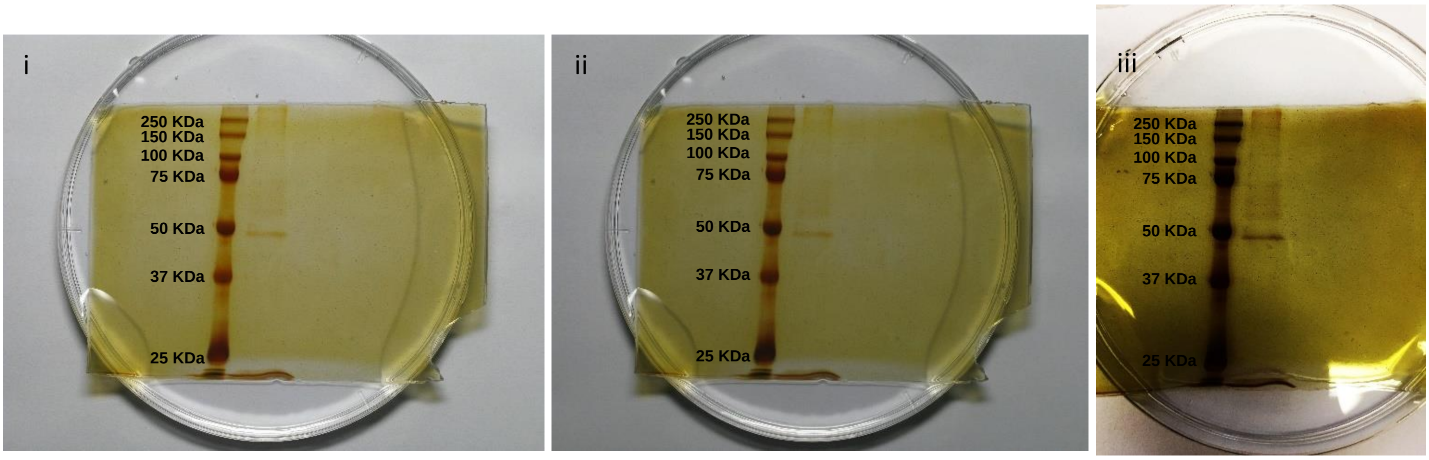

Figure S2: Protein cargo from *A. fumigatus* EVs. Protein content of vesicles was visualized by SDS-PAGE silver stained, according to materials and methods section. Images were acquired using MultiDoc-It Imaging System and Doc-It LS Image Acquisition Software (aperture 5.6; shutter 1/4). Figures i, ii and iii show different expositions from the same SDS-PAGE gel.

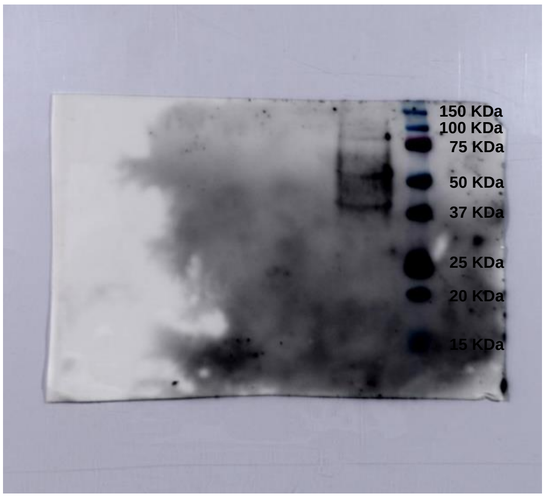

Figure S3: Immunoreactive proteins from *A. fumigatus* vesicles. Proteins from *A. fumigatus* EVs were transferred to nitrocellulose membrane and submitted to immunoblotting according to material and methods section. Total anti-serum from previously infected mice was able to identify some EVs proteins. The image was obtained using Amersham Imager 600 (GE).

Table S1: Proteins identified by proteomic analysis of *A. fumigatus* EVs and classified according to their localization and function.

Protein group number	*A. fumigatus* genome database acession number	Protein name	Localization	Molecular Function	Biological process
1	AFUB_063700	Glutamate dehydrogenase	extracellular region, cytosol, nucleus	oxidoreductase activity	filamentous growth, cellular amino acid metabolic process
2	AFUB_016770	Uncharacterized protein	unknown	hydrolase activity, peptidase activity	proteolysis
3	AFUB_063890	Ecm33	mitochondrion, membrane, cell wall, plasma membrane	unknown	developmental process, cell cycle, response to chemical, sexual sporulation
4	AFUB_096050	Allergen Asp F3 (Peroxiredoxin family protein)	extracellular region, cell wall, peroxisome	oxidoreductase activity	cellular catabolic process, cellular homeostasis, cellular response to oxidative stress, cofactor metabolic process, drug metabolic process, reactive oxygen species metabolic process, regulation of cellular process, response to toxic substance*
5	AFUB_037910	Ubiquitin (UbiC), putative	extracellular region, cytosol, ribosome	structural molecule activity	ribosome biogenesis, organelle organization, cellular protein modification process, translation, RNA metabolic process
6	AFUB_015530	Extracellular cell wall glucanase Crf1/allergen Asp F9	cell wall	hydrolase activity	carbohydrate metabolic process
7	AFUB_066130	Aminopeptidase	extracellular region, cell wall, peroxisome, mitochondrion, endomembrane system, vacuole	hydrolase activity, peptidase activity	peptide catabolic process, proteolysis*
8	AFUB_094680	FAD/FMN-containing isoamyl alcohol oxidase MreA	unknown	oxidoreductase activity	oxidation-reduction process
9	AFUB_094730	IgE-binding protein, putative	unknown	unknown	uknown

10	AFUB_097210	Carboxypeptidase	extracellular region	peptidase activity, hydrolase activity	protein catabolic process, regulation of biological process, conjugation
11	AFUB_095500	GPI anchored protein, putative	cell wall	unknown	uknown
12	AFUB_005920	Glycogenin	cytoplasm*	transferase activity	carbohydrate metabolic process
13	AFUB_002680	Uncharacterized protein	unknown	unknown Carbohydrate binding	uknown
14	AFUB_099560	Tripeptidyl-peptidase (TppA), putative	unknown	hydrolase activity, peptidase activity	proteolysis
15	AFUB_004489	FG-GAP repeat protein, putative	unknown	unknown	uknown
16	AFUB_045170	Cell wall protein phiA	extracellular region	unknown	developmental process, cell cycle, sexual sporulation
17	AFUB_048140	Extracellular phytase, putative	extracellular region	hydrolase activity, phosphatase activity	uknown
18	AFUB_020900	Allergen Asp F4	extracellular region	IgE binding	uknown
19	AFUB_022370	1,3-beta-glucanosyltransferase gel4	extracellular region, cell wall, membrane, plasma membrane	transferase activity	carbohydrate metabolic process
20	AFUB_052010	Nucleoside diphosphate kinase	extracellular region, cytosol, mitochondrion	transferase activity	response to stress, filamentous growth, cellular protein modification process
21	AFUB_052060	Thioredoxin reductase, putative	extracellular region	oxidoreductase activity	response to chemical, secundary metabolic process, toxin metabolic process
22	AFUB_052270	Class III chitinase ChiA1	extracellular region, cell wall, cellular bud neck*	hydrolase activity	carbohydrate metabolic process
23	AFUB_050860	Major allergen Asp F1	unknown	hydrolase activity, RNA binding	regulation of biological process
24	AFUB_052690	Molecular chaperone Mod-E/Hsp90	cell wall, cytosol, membrane, plasma membrane	hydrolase activity, protein binding	response to stress, response to chemical, filamentous growth, regulation of biological process, signal transduction, organelle organization, protein folding, DNA metabolic process, pathogenesis
25	AFUB_085650	Endo-chitosanase	unknown	hydrolase activity	uknown

26	AFUB_046050	Alpha,alpha-trehalose glucohydrolase TreA/Ath1	extracellular region, cell wall, vacuole	hydrolase activity	carbohydrate metabolic process, response to stress, response to chemical
27	AFUB_048180	Probable glucan endo-1,3-beta-glucosidase eglC	cell surface/cell wall*	hydrolase activity, transferase activity	carbohydrate metabolic process
28	AFUB_047510	Extracellular conserved serine-rich protein	unknown	unknown	uknown
29	AFUB_047560	FAD-dependent oxygenase, putative	extracellular region/cell wall	oxidoreductase activity	secundary metabolic process
30	AFUB_037350	Phosphoglucomutase PgmA	cytosol*	isomerase activity	carbohydrate metabolic process, cellular homeostasis
31	AFUB_050510	BYS1 domain protein, putative	unknown	unknown	uknown
32	AFUB_040810	Aspartyl aminopeptidase	extracellular region, vacuole	hydrolase activity, peptidase activity	response to chemical, protein folding
33	AFUB_010890	1,3-beta-glucanosyltransferase Bgt1	extracellular region, cell wall	hydrolase activity, transferase activity	carbohydrate metabolic process, pathogenesis
34	AFUB_023440	60S ribosomal protein L18	cytosolic large ribosomal subunit*	structural constituient of ribosome*	cytoplasmic translation*
35	AFUB_018250	1,3-beta-glucanosyltransferase gel1	extracellular region, cell wall, membrane, plasma membrane	transferase activity	carbohydrate metabolic process, response to chemical, developmental process, response to stress, cell cycle, asexual sporulation, sexual sporulation
36	AFUB_009540	Adenosylhomocysteinase	extracellular region	hydrolase activity	lipid metabolic process, cellular amino acid metabolic process
37	AFUB_066060	GPI anchored cell wall protein, putative	unknown	unknown	uknown
38	AFUB_087520	Isoamyl alcohol oxidase, putative	unknown	oxidoreductase activity	oxidation-reduction process
39	AFUB_006000	40S ribosomal protein S3, putative	extracellular region, cytosol, ribosome	RNA binding, protein binding, structural molecule activity	transport, ribosome biogenesis, translation

40	AFUB_004410	Ubiquitin UbiA, putative	extracellular region, cytosol, ribosome, membrane	structural molecule activity	transport, ribosome biogenesis, translation, organelle organization, cellular protein modification process
41	AFUB_079620	Uncharacterized protein	extracellular region	oxidoreductase activity	oxidation-reduction process
42	AFUB_050490	Glyceraldehyde-3-phosphate dehydrogenase	extracellular region, cytosol, membrane, cell wall, peroxisome, mitochondrion, plasma membrane	RNA binding, oxidoreductase activity, protein binding	carbohydrate metabolic process, transport, regulation of biological process, cell adhesion
43	AFUB_023550	Probable Xaa-Pro aminopeptidase pepP	cytoplasm*	peptidase activity, hydrolase activity	cellular process
44	AFUB_005160	Probable NAD(P)H-dependent D-xylose reductase xyl1	extracellular region	RNA binding, oxidoreductase activity	carbohydrate metabolic process, response to stress, response to chemical
45	AFUB_036480	Putative UDP-galactopyranose mutase	extracellular region, cell wall	isomerase activity	carbohydrate metabolic process, filamentous growth
46	AFUB_034560	Uncharacterized protein	unkown	unknown	uknown
47	AFUB_093550	Actin Act1	peroxisome, cytoskeleton, site polarized growth, action cytoskeleton	structural molecule activity*	cell cycle, endocytosis, exocytosis, intracellular protein transport*
48	AFUB_021670	ER Hsp70 chaperone BiP, putative	extracellular region, membrane, nucleus, endoplasmic reticulum, cell cortex, endomembrane system	hydrolase activity, protein binding	transport, regulation of biological process, carbohydrate metabolic process, response to chemical, organelle organization, protein catabolic process, response to stress, signal transduction, conjugation, nucleus organization
49	AFUB_006770	Elongation factor 1-alpha	extracellular region, mitocondrion, membrane, vacuole	hydrolase activity, RNA binding	transport, translation, organelle organization, cytoskeleton organization
50	AFUB_056780	Superoxide dismutase [Cu-Zn]	extracellular region, cell wall, mitochondrion, cytosol, nucleus	protein binding, oxidoreductase activity	regulation of biological process, response to chemical, response to stress, lipid metabolic process, filamentous growth, developmental process, cellular homeostasis, pathogenesis

51	AFUB_007770	Molecular chaperone Hsp70	extracellular region, cell wall, mitochondrion, membrane, cytosol, nucleus, vacuole, plasma membrane	protein binding, RNA binding	transport, regulation of biological process, response to chemical, organelle organization, protein catabolic process, protein folding
52	AFUB_009760	Phosphoglycerate kinase	extracellular region, cell wall, mitochondrion, membrane, plasma membrane	transferase activity	regulation of biological process, carbohydrate metabolic process
53	AFUB_036860	60S ribosomal protein L22, putative	ribosome	structural molecule activity	translation
54	AFUB_025910	60S acidic ribosomal protein P2/allergen Asp F 8	cytosol, ribosome	structural molecule activity, enzyme regulator activity	regulation of biological process, translation
55	AFUB_017890	Purine nucleoside permease, putative	unknown	transporter activity	transport
56	AFUB_000660	CFEM domain protein	cell wall	unknown	uknown
57	AFUB_089500	Uncharacterized protein	unknown	unknown	uknown
58	AFUB_070900	Uncharacterized protein	unknown	unknown	uknown
59	AFUB_024920	Dipeptidyl-peptidase 5	cytosol, nucleus*	hydrolase activity, peptidase activity	proteolysis
60	AFUB_001190	Ribosomal protein S13p/S18e	extracellular region, ribosome	structural molecule activity, RNA binding	transport, translation, ribosome biogenesis, RNA metabolic process

* represents classification according to, at least, 50% of identity with *Neurospora crassa*, *Saccharomyces cerevisiae* or *Schizosaccharomyces pombe*.
